# A Classic Presentation of Desmoplastic Small Round Cell Tumor

**DOI:** 10.7759/cureus.17582

**Published:** 2021-08-30

**Authors:** Gustavo Matute, Julieth Alexandra Franco Mira, Astrid Arroyave Toro, Fredy Alberto Quintero, Jorge Alberto Bernal, Felipe Solórzano, Néstor Llinás Quintero

**Affiliations:** 1 Pathology, Clinica Medellín, Medellín, COL; 2 Pathology, University of Cartagena, Cartagena, COL; 3 Radiology, Health and Life Imaging, Medellín, COL; 4 Gynecologic Oncology, Colombian Cancer Foundation – Vida Clinic, Medellín, COL; 5 Surgical Oncology, Colombian Cancer Foundation – Vida Clinic, Medellín, COL; 6 Oncology, Colombian Cancer Foundation – Vida Clinic, Medellín, COL

**Keywords:** desmoplastic small round cell tumor, intra-abdominal, immunohistochemistry, wt1, chemotherapy

## Abstract

Desmoplastic small round cell tumor (DSRCT) is a rare malignancy, of uncertain differentiation, which more commonly affects adolescents and young adult males; it usually has an intra-abdominal location. We describe the case of a 35-year-old male who presented initially with occasional abdominal pain, and subsequently with abdominal mass sensation, without any other associated symptoms. Imaging studies reported an intra-abdominal mass located in mesogastrium, right hypochondrium, and right lumbar region, without clear evidence of infiltration to secondary structures, but with clear peritoneal spread to greater omentum and pelvis. Definitive diagnosis of DSRCT is based on histologic and immunohistochemical findings. Standard treatment includes intensive neoadjuvant chemotherapy, surgical cytoreduction, and radiotherapy. Despite this multidisciplinary approach, DSRCT has a poor prognosis and a high mortality rate at five years.

## Introduction

Desmoplastic small round cell tumors (DSRCTs) are highly aggressive malignant tumors characterized by polyphenotypic differentiation [1]. Originally described in 1989 by Gerard and Rosai, these tumor cells were classified as primitive cells with diverse phenotypic differentiation [2]. However, by 1991, DSRCT was formally established as a separate entity [3]. These tumors more frequently occur in young men, with a peak incidence between the second and third decades of life. The mean age at diagnosis is 20.8 years, and it has a male-to-female ratio of 10:1, with no evidence of any ethnic predilection [4,5].

DSRCT usually presents as a single abdominal mass associated with abdominal distention, pain, ascites, or other symptoms due to extrinsic mass effect, or compromise of intra-abdominal organs. However, primary lesions have also been described as arising at other anatomic sites [5]. Imaging studies show multifocal abdominopelvic masses, with extensive serosal involvement, and occasional omental, mesenteric, and retroperitoneal involvement, as well as secondary invasion to viscera. These lesions are usually heterogeneous and exhibit venous contrast enhancement, low attenuation due to central necrosis, bleeding, or fibromyxoid changes. Lymph node involvement is frequent, commonly affecting retroperitoneal, mediastinal, and mesenteric nodes [6]. Macroscopically, multiple nodules of varying sizes can be observed, and a dominant tumoral mass is often identified. The cut surface is firm, grayish-white, and with hemorrhagic and necrotic foci [5].

Histologically, DSRCTs constitute small round or oval neoplastic cells, which are arranged in nests, and surrounded by prominent desmoplastic stroma. Nest size varies considerably and may be accompanied by central necrosis or cystic degeneration [5,6]. The Standard of care for these patients is multidisciplinary, including chemotherapy, cytoreductive surgery (CCS), and radiotherapy; However, in spite of this multidisciplinary approach, the prognosis is poor, and survival rates at three years are reported to be below 50% [5,7].

## Case presentation

A 35-year-old male presented with a history of occasional, low to moderate abdominal pain and distention for about four months, and later with a sensation of an abdominal mass. His past medical history was unremarkable, and he had a family history of hematologic neoplasia in his maternal grandfather. Physical examination showed a patient with obesity, whose vital signs were within normal ranges; the abdomen was soft and non-tender, and a large, fixed, non-tender, intra-abdominal mass in his right lumbar region was palpated. He exhibited no signs of peritoneal irritation. Initial lab tests including complete blood count, ionogram, renal and hepatic function, and tumor markers [Carbohydrate antigen 19-9 (CA19-9): 4.08 U/ml; carcinoembryonic antigen (CEA): 1.19 ng/ml] were within normal ranges.

**Figure 1:**
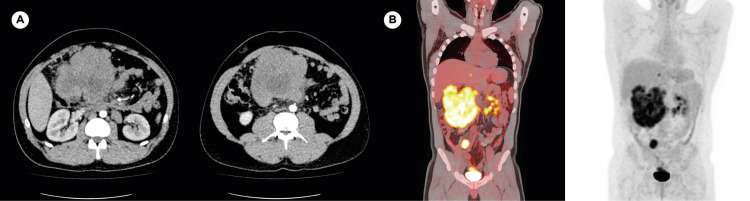
Imaging studies A) Left: contrast CT axial planes show large, heterogeneous mass occupying the epi- and mesogastrium, as well as periaortic and interaortocaval lymphadenopathies. B) Right: PET and fusion (PET-CT) images: coronal plane maximum intensity projection showed a large, lobulated mass in mesogastrium with SUVmax of 9.7, hypodense center due to central necrosis, pelvic implant (posterosuperior to the bladder), and right common iliac metastatic adenopathy CT: computed tomography; PET: positron emission tomography

Contrast-enhanced CT of the abdomen showed a large, heterogeneous, lobulated 11 x 12 x 15-cm mass with contrast enhancement in the periphery with possible central necrosis, in the right upper quadrant and right flank, extending from the mesogastrium to the right lumbar area, in contact but without infiltration of loops of the small bowel and the right rectus muscle. Right paraaortic lymphadenopathy measuring up to 2.9 x 2.1 cm, and right common iliac lymphadenopathy measuring up to 2.3 x 2.9 cm were also identified (Figure 1A). A fused positron emission tomography (PET)-CT scan showed a hypermetabolic mass in the right hemiabdomen, with multiple small satellite hypermetabolic nodules. Multiple enlarged hypermetabolic retroperitoneal and pelvic lymph nodes were also identified. Additionally, hypermetabolic lesions in the right paravertebral, hepatorenal, and pelvic spaces were also identified, most likely representing tumoral extension (Figure 1B).

**Figure 2:**
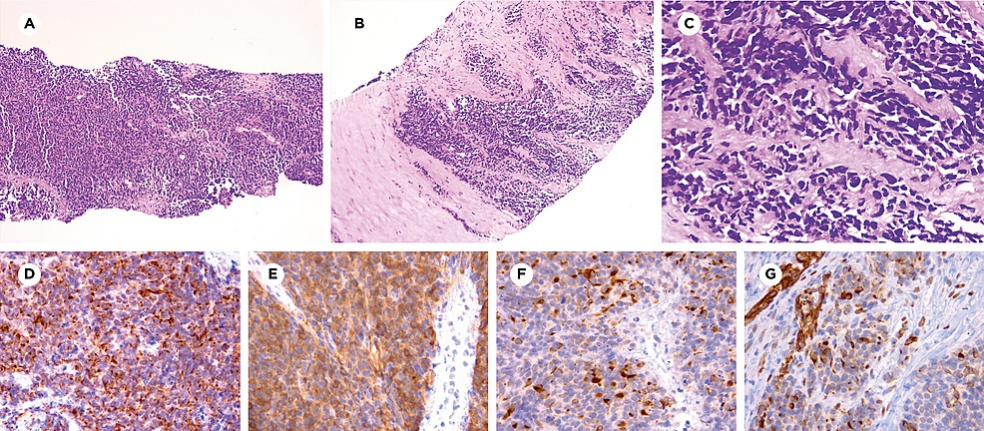
Microscopic features A and B) Malignant neoplasm composed of cells with a diffuse growth pattern, sheets, and nests, which infiltrated the stroma (immersive view and panoramic view, respectively). C) Oval or fusiform cells with eosinophilic cytoplasm and dense nuclei with some mitosis, surrounded by scarce fibrous stroma (400X). D) Cytokeratins (AE1/AE3) (400X). E) Epithelial membrane antigen (EMA) (400X). F) Desmin (400X). G) Wilms tumor 1 protein (WT1) (400X)

CT-guided biopsy was performed, and the pathology report showed malignant neoplasia formed by oval or fusiform cells with eosinophilic cytoplasm, dense nuclei with some mitoses, showing a diffuse growth pattern, in sheets or nests surrounded by fibrous stroma (Figures 2A, 2B). Immunohistochemical studies reported the expression of cytokeratins (AE1/AE3), epithelial membrane antigen (EMA), TLE-1, FLI-1, desmin, CD56, and CD99. Only a few tumoral cells showed nuclear staining for Wilms tumor 1 (WT1) protein. Negative staining was reported for CK7, CK20, synaptophysin, chromogranin, S100, leukocyte common antigen (LCA), h-Caldesmon, myogenin, CD117, and DOG-1. Expression of proliferation index Ki-67 was 70%. Based on all these findings, a diagnosis of DSRCT was made.

The case was discussed during the oncology and gastrointestinal tumor board staff meeting, and the chosen therapeutic approach was systemic therapy with vincristine sulfate, doxorubicin hydrochloride, cyclophosphamide followed by ifosfamide and etoposide (VAC/IE), and according to chemotherapy tolerance and response, a surgical approach would be attempted.

The patient tolerated the first VAC/IE cycle well and subsequently underwent CCS with hyperthermic intraperitoneal chemotherapy (HIPEC). During the surgery, a large soft tissue mass was identified with involvement in the root of the mesocolon, transverse and right colon, part of the distal mesoileum, and there was an attachment to the root of the superior mesenteric artery and vein. In addition, peritoneal carcinomatosis and nodular lesions in the greater omentum, and a 4 x 3-cm lesion in the cul-de-sac were identified. Furthermore, there was also the involvement of mesocolic, the root of the small bowel, and para-aortic lymph nodes.

Complete cytoreduction was achieved (completeness of cytoreduction score: CC-0), including paraaortic, pelvic, and mesenteric lymph node dissection. HIPEC was administered using the closed technique (cisplatin 100 mg/m^2^ for 90 minutes at 41 °C). No intraoperative complications were reported. Postoperative recovery was acceptable; however, the patient presented acute kidney failure due to cisplatin toxicity as evidenced by an increased creatinine level (2.6 mg/dL), despite which no dialysis was required.

Specimens received by the pathology laboratory included right hemicolectomy and dissected lymph nodes corresponding to mesenteric, left para-aortic, left pelvic, right pelvic, and interaortocaval nodes. Histologic sections showed malignant neoplasia similar to that described in the biopsy results, and of a total of 41 dissected lymph nodes, 19 showed evidence of neoplastic disease. Tumor implants were identified in the interaortocaval dissection and in the pelvic soft tissue.

## Discussion

Desmoplastic small round cell tumor (DSRCT) is a soft tissue malignant neoplasm of uncertain differentiation. It belongs to the small round cell tumor family, which also includes tumors such as neuroblastoma, rhabdomyosarcoma, Wilms tumor (WT), and Ewing sarcoma (EWS) family of tumors [1]. Due to a lack of distinctive morphological features differentiating these tumors, immunohistochemical studies and genetic studies are required to reach a specific diagnosis and decide on a therapeutic regimen [1].

Initially described in 1989 by Gerard and Rosai, DSRCT tumor cells were classified by their morphologic and immunologic features as primitive cells with multiphenotypic differentiation, since they were found to express epithelial, neuronal, skeletal muscle, and mesenchymal markers [2]. However, by 1991, DSRCT was formally established as a separate clinicopathological entity [3]. It has a predilection for young men, with ages ranging from three to 52 years, and with a peak incidence between the second and third decades of life. The mean age at diagnosis is 20.8 years, and it has a male-to-female ratio of 10:1, with no evidence of any ethnic predilection [4,5].

Clinically, there are no specific symptoms, and most patients present with a single abdominal mass, as in our case. It is sometimes associated with pain, abdominal distention and/or ascites, constipation, weight loss, or other symptoms secondary to extrinsic mass effect (bowel obstruction) [8] or due to compromise of abdominopelvic organs [4,5].

The classical location of DSRCT is intra-abdominal, and while its histogenesis is unknown, a possible mesothelial origin has been proposed based on the predominant involvement of serosae including parietal and visceral peritoneum, pleura, and tunica vaginalis in paratesticular lesions (continuity of peritoneum) [4,9]. Nonetheless, a few reports have described primary lesions in other locations such as the intracranial, sinonasal cavity, salivary glands, as well as colonic, renal, and soft tissue [6].

Imaging studies often identify multifocal abdominopelvic masses with extensive serosal involvement, and occasionally, the involvement of omentum, mesentery, retroperitoneum, and secondary visceral invasion (pancreas, spleen, and kidney) [10,11]. A dominant mass, which is at least double in size of other lesions, can be identified in up to 88% of patients. These masses are usually heterogeneous and show venous contrast enhancement, low attenuation due to central necrosis, bleeding, or fibromyxoid changes in up to 50% of cases, and calcifications in up to 25% of cases [10,11]. At the time of diagnosis, lymph node involvement is frequent in up to 50% of the patients, with retroperitoneal nodes (88%) being the most commonly involved, followed by mediastinal (25%), and mesenteric (14%) lymph nodes. In addition, low-volume ascites and pleural effusion can also be found [10,11].

The frequency of metastatic lesions ranges from 25% to 50%, being more commonly found in the liver and bone. MRI may be useful when surgical management is being considered to better define the disease extension since these tumors exhibit strong signal intensity on T2-weighted sequences while being hypo or isointense relative to skeletal muscle on T1-weighted sequences and enhancement following administration of venous contrast [11]. On PET-CT, desmoplastic tumors typically exhibit intense fluorodeoxyglucose (FDG) uptake, and hence PET-CT could potentially play a role in the initial evaluation and follow-up (early detection of relapse) [11]. Given its location, size, and aggressive behavior, differential diagnosis of imaging findings include malignant peritoneal mesothelioma, rhabdomyosarcoma, lymphoma, leiomyosarcoma, and disseminated malignant disease (carcinomatosis and sarcomatosis) [5,12].

Macroscopically, multiple nodules of varying size, and often a dominant, large (maximum tumor diameter of up to 50 cm has been reported), lobulated, white, tumoral mass can be observed. The cut surface is firm, grayish-white in color, and with hemorrhagic foci and occasional cystic degeneration [5,12].

Histologically, DSRCTs constitute small neoplastic cells, which are relatively uniform, whose size and shape can be varied (epithelioid, oval, fusiform), with hyperchromatic nuclei, unremarkable nucleoli, and scarce eosinophilic cytoplasm. Intracytoplasmic inclusions and even rhabdoid features can be observed in up to 50% of tumor cells [13]. These cells are predominantly arranged in well-defined nests of varying size, as well as in cords, sheets, trabeculae, solid areas, rosettes, and/or pseudo-rosettes [9], with extensive necrosis, frequent mitotic figures, cystic degeneration and/or calcification, surrounded by a prominent desmoplastic stroma composed of fibroblasts or myofibroblasts, with loose extracellular material and abundant collagen [5,12]. These morphologic features suggest as differential diagnoses EWS, WT, rhabdomyosarcoma, non-Hodgkin’s lymphoma, synovial sarcoma, leiomyosarcoma, and peritoneal carcinomatosis [5,13].

The immunohistochemical profile of these neoplastic cells has been described as a variable co-expression of epithelial, mesenchymal, neuronal, and skeletal muscle markers [2,5]. Epithelial markers cytokeratins (AE1/AE3, CAM5.2) are expressed in about 70% and can show positivity diffuse or dot expression; EMA is expressed in about 90% of DSRCTs [5]. Regarding mesenchymal markers, positive expression of desmin has been reported in up to 72% of lesions, and the expression is typically in a para-nuclear dot distribution or diffuse; while neuronal markers (S100, chromogranin, synaptophysin) and muscle markers (h-Caldesmon, myogenin) have been reported as negative [5]. In agreement with these findings, our patient’s specimens were positive for EMA, and cytokeratins markers expressed a diffuse patron and desmin marker showed a dot expression while being negative for S100, chromogranin, synaptophysin, h-Caldesmon, and myogenin. In addition, samples showed a positive expression of WT1 protein, which has been previously reported to be expressed in over 90% of cases [14].

Additional differential diagnosis markers, such as TLE-1, FLI-1, CD56, and CD99, showed positive expression, while CK7, CK20, LCA, CD117, and DOG-1 were negative (Table 1) [1,2,5]. Therefore, while the histogenesis of this tumor remains to be fully understood, previous studies have hypothesized that DSRCT might be derived from either multipotent primitive mesenchymal cells, primitive mesenchymal, or neuroectodermal tissue [7].

**Table 1:** Patient immunohistochemical markers compared with markers expressed in desmoplastic small round cell tumors *Adapted from Thway, et al. [5] AE1/AE3: cytokeratin AE1/AE3; EMA: epithelial membrane antigen; TLE-1: transducin-like enhancer of split 1; FLI-1: friend Leukemia virus integration site 1; CD99: cluster of differentiation 99 or MIC2 or single-chain type-1 glycoprotein; WT1: Wilms tumor 1 protein; CK7: cytokeratin 7; CK20: cytokeratin 20; CLA: common leukocyte antigen; S100: S100 protein; DOG-1: discovered on GIST

Markers	Case	Reported percentage of positive expression*
AE1/AE3	+	70-80%
EMA	+	90%
TLE-1	+	Not reported
FLI-1	+	Not reported
Desmin	+	90%
CD56	+	>80%
CD99	+	>50%
WT1	+	>90%
CK7	-	Negative
CK20	-	Negative
Synaptophysin	-	15%
Chromogranin	-	<5%
S100	-	<10%
CLA	-	Negative
h-Caldesmon	-	Not reported
Myogenin [2]	-	80%
CD117	-	Not reported
DOG-1	-	Not reported

Cytogenetic studies describe an association with reciprocal chromosomal translocation t(11;22)(p13;q11 or p13;q12), in which exon 7 of the EWS gene and exon 8 of the WT gene are fused, producing a chimeric protein (EWT-WT1) with transcriptional activity related to oncogenic activity since it leads to the expression of platelet-derived growth factor subunit A (PDGF-A), interleukin 2 beta receptor (IL-2RB), epidermal growth factor (EGF), insulin-like growth factor 1 (IGF-1), and myeloid leukemia factor 1 (MLF-1), which play a role in stimulating fibroblastic proliferation, a relevant feature in DSRCTs. While the EWS-WT1 fusion gene has been described as a genetic mutation specific for DSRCTs, it has also been recently described in small round cell tumors of the cauda equina [5,14]. The EWT-WT1 fusion gene can be detected by reverse transcription-polymerase chain reaction (RT-PCR) or by fluorescent in situ hybridization (FISH) with a sensitivity of 93% and 97%, respectively, and a specificity of 100% for RT-PCR [5,14].

Therapeutic management of DSRCT is challenging since it is a rare and aggressive tumor. The current standard of care requires a multidisciplinary approach that must be tailored to each patient [5]. Intensive chemotherapy with agents used to treat EWS and the VAC/IE regimen have been previously used to treat DSRCTs [15], which was also the initial regimen of choice for our patient.

Aggressive surgical cytoreduction in cases of intra-abdominal, single lesions, without evidence of metastasis has been shown to lead to increased three-year survival of up to 58% when complete cytoreductive resection has been achieved [7]. Complete surgical cytoreduction and HIPEC, also known as the Sugarbaker technique, have been described among the therapeutic approaches used to achieve complete tumor resection, as well as the removal of peritoneal tumor implants and lymph node dissection [16]. In one of the largest case series reported to date, which included 187 patients at The University of Texas MD Anderson Cancer Center, DSRCT treatment with systemic chemotherapy and complete CCS was associated with improved overall survival [16]. Therefore, our patient underwent received systemic chemotherapy, CCS, and HIPEC. He is currently receiving adjuvant systemic therapy with VAC/IE regimen, with adequate tolerance.

Total abdominopelvic radiotherapy (WAP-RT) is a complementary treatment option that has been used with variable results in patients with DSRCT; although new radiotherapy techniques such as intensity-modulated radiation therapy (IMRT) have not achieved a statistically significant improvement in patient survival, they have shown a significant decrease in toxicity, improving tolerance in patients when used as a therapeutic modality after chemotherapy and CCS [5,9]. Despite all the efforts to improve its treatment, DSRCTs still have a poor prognosis, with three- and five-year survival rates below 50% and 20%, respectively [5,7,17].

## Conclusions

DSRCT is a highly aggressive and rare neoplasm that has been classified as a soft tissue sarcoma, belonging to the small round cell tumor family. Clinical symptoms are non-specific, and the condition should be suspected in young patients with imaging reports of multiple peritoneal lesions or a single intra-abdominal mass without a clear association to abdominopelvic viscera. The current diagnostic gold standard for DSRCT includes histopathologic, immunohistochemical, and cytogenetic studies in order to confirm the variable phenotypic expression and characteristic chromosomal translocation.
